# Evidence of *Orientia* spp. Endemicity among Severe Infectious Disease Cohorts, Uganda

**DOI:** 10.3201/eid3007.231040

**Published:** 2024-07

**Authors:** Paul W. Blair, Kenneth Kobba, Stephen Okello, Sultanah Alharthi, Prossy Naluyima, Emily Clemens, Hannah Kibuuka, Danielle V. Clark, Francis Kakooza, Mohammed Lamorde, Yukari C. Manabe, J. Stephen Dumler, Acute Febrile Illness, Sepsis in Uganda study teams

**Affiliations:** Uniformed Services University, Bethesda, Maryland, USA (P.W. Blair, S. Alharthi, E. Clemens, J.S. Dumler);; Henry M. Jackson Foundation for the Advancement of Military Medicine, Inc., Bethesda (P.W. Blair, S. Alharthi, D.V. Clark);; Johns Hopkins University School of Medicine, Baltimore, Maryland, USA (P.W. Blair, Y.C. Manabe);; Infectious Diseases Institute, Makerere University, Kampala, Uganda (K. Kobba, F. Kakooza, M. Lamorde);; Makerere University Walter Reed Project, Kampala (S. Okello, P. Naluyima, H. Kibuuka)

**Keywords:** Scrub typhus, Orientia, bacteria, vector-borne infections, acute febrile illness, Uganda, Africa

## Abstract

At 3 severe infection cohort sites in Uganda, *Orientia* seropositivity was common. We identified 4 seroconversion cases and 1 PCR-positive case. These results provide serologic and molecular support for *Orientia* spp. circulating in sub-Saharan Africa, possibly expanding its endemic range. *Orientia* infections could cause severe illness and hospitalizations in this region.

Scrub typhus is a leading cause of nonmalarial febrile illness in Southeast Asia (*1*). Scrub typhus is caused by miteborne *Orentia tsutsugamushi* infections, which until recently were thought to be limited to South and Southeast Asia. Molecular identification of different *Orientia* species in clinical cases from Chile (*2*) and the United Arab Emirates (*3*) has suggested a broader epidemiology. *Orientia* spp. were found in mites in Kenya (*4*), and descriptions of *Orientia* seroconversion in patients from sub-Saharan Africa have slowly accrued, suggesting the possibility of *Orientia* spp. transmission in Africa (*5*). We used archived samples collected in 2 severe infection prospective cohorts in western, central, and northwest Uganda to assess *Orientia* endemicity in the country. 

## The Study

Using archived samples, we measured serial *Orientia* immunofluorescence assay (IFA) IgG titers and performed reflex *Orientia* spp. reverse transcription PCR (RT-PCR). Samples were collected as part of 2 severe infection prospective cohorts and had undergone broad microbiologic testing. In both cohorts, adult patients >18 years of age who fulfilled acute febrile illness (AFI; hospitals in Mubende and Arua, Uganda) or sepsis-specific (hospital in Fort Portal, Uganda) eligibility criteria were evaluated for enrollment at admission in the outpatient or emergency department, or on medical wards (Appendix) (*6*). Matched acute and convalescent serum samples were available from 269 of 310 participants enrolled in the sepsis cohort and 67 of 132 participants in the AFI cohort.

In brief, across both prospective cohorts, study teams collected demographic and symptom information, examination findings, and laboratory data on standardized forms during hospitalization and at 1 month after enrollment. Clinical tests were routinely performed, including complete blood counts and chemistries. Microbiologic testing included blood culture with antimicrobial sensitivity testing, HIV testing, malaria smears, and rapid diagnostic tests, as previously described (*6*) (Appendix).

 We performed IgG IFAs by using *Orientia tsutsugamushi* Karp strain antigen slides (BIOCELL Diagnostics Inc., https://biocelldx.com). Baseline (acute) and 1-month follow-up (convalescent) serum samples were screened at a titer of 1:64 and titrated up to 1:65,000. We considered a sample seropositive at a threshold titer of >128. We performed IgG IFAs by using commercial slides to evaluate for cross-reactivity to spotted fever group rickettsia (SFGR), *Rickettsia conorii* Molish 7 strain, typhus group rickettsia (TGR), and *Rickettsia typhi* Wilmington strain (BIOCELL Diagnostics, Inc.). We performed a Kruskal-Wallis test to evaluate for differences between *Orientia* IFA IgG titers between those with and without available matched samples. We used a titer of 32 to calculate -fold increase if the screen was negative at a titer of 1:64. We had a blind second reader review <5% of each batch. 

Because no prior estimates of *Orientia* seroprevalence were available for Uganda, we used stringent criteria to define probable cases (Appendix Figure 1). To evaluate the specificity of IFA results, we used a subset of high titer samples to corroborate evidence of antibody binding by using a dot blot, Western blot, and Gilliam strain IFA (Appendix Methods, Figure 2). To optimize sensitivity for RT-PCR, we targeted mRNA and rRNA from serum from both cohorts (*7*), whole blood from the AFI cohort, or buffy coat from the sepsis cohort. We used QIAamp RNA Mini Kit (QIAGEN, https://www.qiagen.com) to extract RNA. We performed RT-PCR targeting *Orientia* spp. 16S rRNA, Orien16S and *rrs* by using previously published methods (*3*,*8*), and mRNA from *Orientia* spp. 56-kDa antigen gene, SFGR OmpA (*sca0*) gene, and TGR kDa (*9*) outer membrane protein gene. We only called positives that were in duplicate.

**Figure 1:**
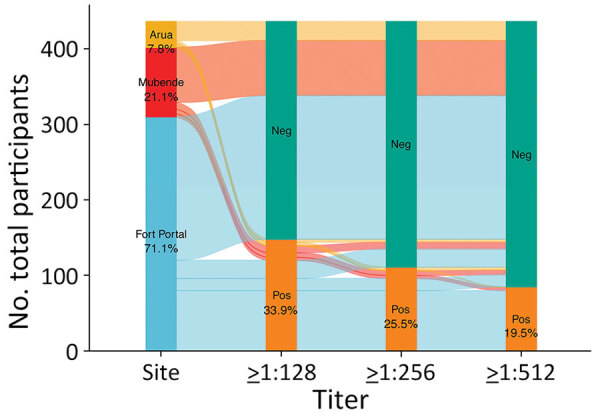
Alluvial diagram of serology from acute serum samples used in a study of *Orientia* genus endemicity among severe infectious disease cohorts, Uganda. The diagram represents *Orientia* spp.–positive immunofluorescent assay IgG at >128, ­>256, and >512 from 3 sites in Uganda. Colored lines indicate total participants from each site with positive or negative serology at 3 different titer cutoffs. Neg, negative; pos, positive.

**Figure 2:**
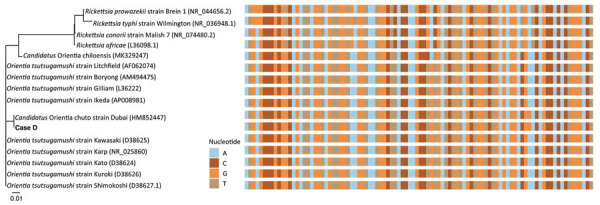
Phylogenetic tree (left) and aligned sequences (right) of *Orientia* spp. and locally endemic *Rickettsia* spp. in a study of *Orientia* genus endemicity among severe infectious disease cohorts, Uganda. We compared the 16S rRNA gene with an *Orientia* infection (case D) in Uganda. We aligned the 96-bp amplicon region and created the tree by using the neighbor-joining algorithm in R (The R Foundation for Statistical Computing, https://www.r-project.org). GenBank accession numbers of reference sequences are in parentheses. A single polymorphism aligned with *Candidatus* O. chuto, possibly differentiating case D from other *Orientia* spp. Scale bar indicates nucleotide substitutions per site.

We found that 33.9% (148/436) of acute samples and 38.4% (129/336) of convalescent samples were seropositive (>128) for *Orientia* spp. Among acute samples, 25.5% (111/436) were positive at >256 titer and 19.0% (85/436) were positive at ≥512 (Figure 1). We observed no difference in acute IFA titers between patients with and without a convalescent blood samples (p = 0.33). Among samples with a positive 1:64 titer screen, the median acute titer was 128 (up to 8,192; interquartile range [IQR] 64­–512) and median convalescent titer was 256 (up to 4,096; IQR 64–1,024). Seropositivity was highest (acute, 38.7% [120/310]; convalescent, 41.6% [112/269]) in Fort Portal, but was also high in Arua (acute, 26.5% [9/34]; convalescent, 30.0% [6/20]) and Mubende (acute, 20.7% [19/92]; convalescent, 23.4% [11/47]). The overall geometric mean titers were 90.8 (95% CI 80.2–102.8) for acute samples and 100.3 (95% CI 86.1–116.9) for convalescent samples.

Four participants met our case definition for *Orientia* spp. seroconversion (Table 1). Participants meeting the case definition were 24–56 years of age; 3 were female and 1 was male, and 3 had HIV (Table 2). Leukocyte counts ranged from 5–10 × 10^3^ cells/μL, platelet counts were 56–220 × 10^3^ cells/μL, and aspartate transaminase was 21–136 U/L. Three patients survived, but a 34-year-old woman with HIV in whom a papular rash developed died of unknown causes 8 months after follow-up. Three participants with seroconversion had negative malaria smears, blood cultures, and rapid antigen and molecular diagnostic tests for nonrickettsial pathogens (Table 2).

**Table 1:** Rickettsia IgG results from participants with *Orientia spp.* seroconversion in a study of *Orientia* genus endemicity among severe infectious disease cohorts, Uganda*

Participant	Days after illness onset†			*Orientia* spp.–positive titer‡				Spotted fever group titer				Typhus group titer		
	Acute	Conv.		Fold change	Acute	Conv.		Fold change	Acute	Conv.		Fold change	Acute	Conv.
Mubende														
A	7	34		4	256	1,024		1	32	32		1	32	32
Fort Portal														
B	2	32		8	64	512		1	32	32		1	32	32
C	14	36		4	128	512		1	32	32		1	32	32
D	1	38		4	128	512		1	32	32		1	32	32

*Conv., convalescent; IFA, immunofluorescent assay. †Sample collection after illness onset. ‡Karp strain IFA. Gilliam strain IFA seroconversion also observed in Fort Portal participant D (acute titers 1:512 and convalescent titers 1:2,048) but not among participants A-C.

**Table 2:** Clinical characteristics of participants with *Orientia spp.* seroconversion in a study of *Orientia* genus endemicity among severe infectious disease cohorts, Uganda*

Characteristics	Patient identification			
	A	B	C	D
Age, y/sex	24/M	34/F	23/F	56/F
Occupation	Mine worker	Business or trade	Fuel attendant	Farmer
Rash, type	Y, pustular and eschar	Y, papular	N	N
Clinical laboratory parameters				
WBC, x 10^3^ cells/μL	7	10	5	8
Platelet count, x 10^3^ cells/μL	56	220	128	177
AST, U/L	21	62	136	26
Microbiologic results†				
HIV (CD4)	+ (603)	+ (NA)	+ (NA)	­–
Malaria smear	+, 126 parasites/μL	–	–	–
TB				
PCR	NA	NA	NA	–
Urine LAM	+	–	–	NA
Clinical diagnosis	TB	Urinary tract infection	Unidentified	Abdominal source
Inpatient treatment	ACT	CIP, CTX, MTZ	CTX, cefixime	CIP, MTZ
Outcome	Survived	Died, 8.2 mo.	Survived	Survived
*ACT, artemisinin-based combination therapy; AST, aspartate transaminase; CIP, ciprofloxacin; CTX, ceftriaxone; LAM, lipoarabinomman; MTZ, metronidazole; NA, not available; NG, no growth; TB, tuberculosis; WBC, white blood cells; –, negative; +, positive. †All had negative blood cultures and negative multiplex PCR results.				

We used molecular methods to confirm *Orientia* spp. infection. The acute serum sample from participant D was repeatedly *rrs*-positive with RT-PCR (mean cycle threshold 34.1, SD 0.4) and was confirmed by Sanger sequencing of the amplicon. A BLAST analysis (https://blast.ncbi.nlm.nih.gov) of a 96-bp sequenced fragment of the amplicon revealed 96%–100% homology with *Orientia* spp., and a single polymorphism aligned with *Candidatus* O. chuto (Figure 2). RT-PCR was negative using other primers for *Orientia* spp. (Orien16S 56-kDa) targets, SFGR (*sca0* [*ompA*] targets, and TGR (17-kDa antigen gene) targets.

## Conclusions

We identified *Orientia* seroconversion among 4 participants hospitalized with severe infection in sub-Saharan Africa. We demonstrated that *Orientia* seropositivity was common among patients admitted for severe infection at 3 hospitals in Uganda. Our findings of highly prevalent seropositivity at 3 sites, identification of seroconversion, and molecular confirmation of a case with otherwise negative broad microbiologic testing support *Orientia* circulation and raise suspicion for infections extending to East Africa. 

Prior clinical evidence of suspected scrub typhus in Africa relied on case reports of returning travelers with *Orientia* seroconversion (*5*). In addition to seroconversion identified in this study, seroconversions were observed in a pediatric cohort in Kenya (3.6%; n = 10) (*10*), and in 1 case among 49 abattoir workers in Djibouti (*11*). Our well-characterized multisite results supplement the limited literature suggesting *Orientia* spp. infections in sub-Saharan Africa. 

In addition to prior suggestive evidence, our results build on a shift in understanding of worldwide *Orientia* spp. clinical infections. SFGR and TGR test results were negative in our cohorts, decreasing the likelihood of cross-reactivity. Despite IFA being the preferred method for rickettsial diagnosis, intrinsic interobserver variability limitations exist (*12*); we aimed to reduce those limitations through our reading approach and seroconversion criteria. Although we were able to confirm an infection by using real-time RT-PCR, sequence results were limited to a small fragment of the abundant 16S rRNA. The clinical relevance requires further confirmation with *Orientia* culture growth and extended genome sequencing. Because we relied on convalescent serology, we might have missed early fatal cases, which could skew our results toward less severe illness. Research efforts are needed to characterize the circulating species, incidence, pathogenic potential, and clinical relevance of *Orientia* infections in East Africa. 

In summary, our findings suggest *Orientia* spp. circulation within the human–environment interface in Uganda and suggest novel *Orientia* infections within severe infection cohorts in Uganda. After excluding common causes of infections, our findings provide evidence of locally acquired *Orientia* infections among adults in sub-Saharan Africa.

Appendix Additional information on evidence of *Orientia* genus endemicity among severe infectious disease cohorts, Uganda.

## References

[1] Bonell A, Lubell Y, Newton PN, Crump JA, Paris DH. Estimating the burden of scrub typhus: A systematic review. PLoS Negl Trop Dis. 2017;11:e0005838. 10.1371/journal.pntd.0005838 28945755 PMC5634655

[2] Weitzel T, Dittrich S, López J, Phuklia W, Martinez-Valdebenito C, Velásquez K, et al. Endemic scrub typhus in South America. N Engl J Med. 2016;375:954–61. 10.1056/NEJMoa1603657 27602667

[3] Izzard L, Fuller A, Blacksell SD, Paris DH, Richards AL, Aukkanit N, et al. Isolation of a novel *Orientia* species (*O. chuto* sp. nov.) from a patient infected in Dubai. J Clin Microbiol. 2010;48:4404–9. 10.1128/JCM.01526-10 20926708 PMC3008486

[4] Masakhwe C, Linsuwanon P, Kimita G, Mutai B, Leepitakrat S, Yalwala S, et al. Identification and characterization of *Orientia chuto* in trombiculid chigger mites collected from wild rodents in Kenya. J Clin Microbiol. 2018;56:e01124–18. 10.1128/JCM.01124-18 30282787 PMC6258837

[5] Richards AL, Jiang J. Scrub typhus: historic perspective and current status of the worldwide presence of *Orientia* species. Trop Med Infect Dis. 2020;5:49. 10.3390/tropicalmed5020049 32244598 PMC7344502

[6] Blair PW, Kobba K, Kakooza F, Robinson ML, Candia E, Mayito J, et al. Aetiology of hospitalized fever and risk of death at Arua and Mubende tertiary care hospitals in Uganda from August 2019 to August 2020. BMC Infect Dis. 2022;22:869. 10.1186/s12879-022-07877-3 36411415 PMC9680122

[7] Yun NR, Kim CM, Kim DY, Seo JW, Kim DM. Clinical usefulness of 16S ribosomal RNA real-time PCR for the diagnosis of scrub typhus. Sci Rep. 2021;11:14299. 10.1038/s41598-021-93541-w 34253778 PMC8275794

[8] Jiang J, Martínez-Valdebenito C, Weitzel T, Farris CM, Acosta-Jamett G, Abarca K, et al. Development of a new genus-specific quantitative real-time PCR assay for the diagnosis of scrub typhus in South America. Front Med (Lausanne). 2022;9:831045. 10.3389/fmed.2022.831045 35573006 PMC9095740

[9] Reller ME, Dumler JS. Optimization and evaluation of a multiplex quantitative PCR assay for detection of nucleic acids in human blood samples from patients with spotted fever rickettsiosis, typhus rickettsiosis, scrub typhus, monocytic ehrlichiosis, and granulocytic anaplasmosis. J Clin Microbiol. 2020;58:e01802–19. 10.1128/JCM.01802-19 32493778 PMC7448621

[10] Maina AN, Farris CM, Odhiambo A, Jiang J, Laktabai J, Armstrong J, et al. Q fever, scrub typhus, and rickettsial diseases in children, Kenya, 2011–2012. Emerg Infect Dis. 2016;22:883–6. 10.3201/eid2205.150953 27088502 PMC4861507

[11] Horton KC, Jiang J, Maina A, Dueger E, Zayed A, Ahmed AA, et al. Evidence of rickettsia and *Orientia* infections among abattoir workers in Djibouti. Am J Trop Med Hyg. 2016;95:462–5. 10.4269/ajtmh.15-0775 27273647 PMC4973201

[12] Phetsouvanh R, Thojaikong T, Phoumin P, Sibounheuang B, Phommasone K, Chansamouth V, et al. Inter- and intra-operator variability in the reading of indirect immunofluorescence assays for the serological diagnosis of scrub typhus and murine typhus. Am J Trop Med Hyg. 2013;88:932–6. 10.4269/ajtmh.12-0325 23478577 PMC3752761

