# Supplementary material for: Evidence of Orientia spp. Endemicity among Severe Infectious Disease Cohorts, Uganda
Source: Emerg Infect Dis. 2024 Jul;30(7):1442–6. doi: 10.3201/eid3007.231040 (PMC11210639; doi:10.3201/eid3007.231040)
Supplement: Appendix — Additional information on evidence of Orientia genus endemicity among severe infectious disease cohorts, Uganda. [file 23-1040-Techapp-s1.pdf]

Article DOI: https://doi.org/10.3201/eid3007.231040

EID cannot ensure accessibility for supplementary materials supplied by authors. Readers who have difficulty accessing supplementary content should contact the authors for assistance.

Evidence of *Orientia* Genus Endemicity among Severe Infectious Disease Cohorts, Uganda

Appendix

Supplemental Methods

Acute Febrile Illness Cohort

As previously described (*1*), in the acute febrile illness cohort, patients presenting to Mubende and Arua Regional Referral Hospitals (RRHs) with a measured or reported temperature ≥38.0°C occurring within the past 48 hours or a clinical history consistent with fever within 48 hours of presentation were enrolled from August 2019 to August 2020 (*1*). Participants with COVID-19 diagnoses were in separate designated areas and were not enrolled. Exclusion criteria included being hospitalized for ≥72 hours, receipt of antibiotics, previous participation in the study, being a prisoner, and being a psychiatric patient. The two tertiary care hospitals were chosen as study sites because both sites are in regions with different ecology and serve different populations. Mubende RRH (bed capacity 173) is located in central Uganda and serves a district population of over 688,000 people (*2*). Arua RRH (bed capacity 323) is located in the more arid northwest Uganda and serves over 785,000 people in the districts.

Sepsis Cohort

Briefly, in the sepsis cohort, 301 patients ≥18 years of age with suspected infection and ≥2 modified systemic inflammatory response syndrome (SIRS) criteria (temperature <36°C or >38°C, heart rate ≥90 beats per minute, or respiratory rate ≥20 breaths per minute) were enrolled at Fort Portal Regional Referral Hospital in Fort Portal, Uganda from October 2017 to November 2021 (P.W. Blair et al., unpub. data, https://doi.org/10.1101/2023.09.14.23295526). Patients presenting at the outpatient department, emergency department, or medical wards were evaluated

for enrollment. Patients were not eligible if they were deemed too ill to participate with an imminently terminal comorbidity or if they presented with severe anemia (hemoglobin <7 g/dL). Due to inflammation biomarker objectives, participants were excluded with known immunocompromising conditions, including drug induced immunosuppression, anatomic or functional asplenia, recent chemotherapy, pregnancy and <6 weeks postpartum females, but participants with HIV were eligible. FPRRH serves as a health facility to eight districts in western Uganda.

Sepsis and AFI Cohort

If there was clinical suspicion, PCR testing for tuberculosis was performed using expectorated sputum (Xpert MTB/RIF Ultra, Cepheid, Sunnyvale, CA, USA) and participants with HIV had a urine lipoarabinomman (LAM test). Whole blood was run on the FilmArray Global Fever panel for 19 non-rickettsial pathogen targets (*3*).

Seroconversion Criteria

To validate the findings of seropositivity, we also read Gilliam strain IFA slides (BIOCELL Diagnostics Inc., Baltimore, MD, USA) for a portion (n = 50), including those that seroconverted and had a >1:256 titer (n = 13) and also 37 (12%) randomly selected samples. We used seroconversion criteria to decrease the risk of a false Karp IFA seroconversion. First, we included seroconversions defined as a ≥4-fold increase in titer from acute to convalescent sera resulting in a convalescent titer of at least 512, excluding n = 4 at 256 (the upper interquartile range [IQR] of the *Orientia* IgG titers). Second, while serum cross-reactivity with spotted fever group rickettsia (SFGR) or typhus group rickettsia (TGR) has not been widely reported (*4*), we excluded participants with convalescent SFGR (n = 7) or TGR (n = 2) IFA IgG titers that were greater than or equal to *Orientia spp.* (SFGR *Rickettsia conorii* Molish 7 strain, TGR *Rickettsia typhi* Wilmington strain; BIOCELL Diagnostics Inc, Baltimore, MD, USA). Third, we excluded those with severe symptoms present for >14 days at time of enrollment (n = 2).

Western Blot and Dot Blot

For the Western blot and dot blot, we used the TSA 56-kDa *Orientia* protein (*5*) to determine existence of *Orientia* spp. antibodies from the serum sample. The recombinant protein was received frozen after preparation using the referenced procedures (*5*) but was dissolved in 8 M urea. The protein was dialyzed to remove urea using Amicon Ultra 0.5 mL centrifugal filter

units (Merck), following established procedures. Centrifugation occurred at 1,000 to 2,000 g for ten minutes and was followed by a wash step with phosphate-buffered saline (PBS, pH 7.4) and an additional centrifugation. The dialyzed protein was carefully collected from the filter units and stored in PBS. Positive controls included *Orientia tsutsugamushi* IgG–positive serum and a healthy unexposed human serum negative control.

For the Western blot, recombinant TSA56 protein was prepared in two concentrations, 2 μg and 5 μg, in NuPAGE LDS Sample Buffer and NuPAGE Sample Reducing Agent (Invitrogen, Thermo Fisher Scientific). The samples underwent denaturation at 70°C for 10 minutes using a heat block (Thermo Fisher Scientific) and were loaded onto a NuPAGE 10% Bis-Tris gel (Invitrogen, Thermo Fisher Scientific). Electrophoresis was conducted at 200V. The proteins were transferred to a membrane using the Trans-Blot Turbo Transfer System (Bio-Rad). Transfer efficiency was assessed by Ponceau S staining (Sigma-Aldrich). The membrane was blocked with 5% non-fat dry milk in PBST (PBS + 0.1% Tween-20) and then incubated with human serum samples diluted 1:100,000, including a positive control serum containing scrub typhus IgG and a negative control of normal human serum. The membrane was treated with HRP-conjugated anti-human IgG (Invitrogen), diluted to 1:2,000. Subsequently, to assess protein purity, the TSA56 protein weight was estimated using ImageJ software (*6*). After $\log_{10}$ transformation to fit a standard curve, the weight of the TSA56 protein was estimated to be 59.8 kDa and, accordingly, within range of anticipated measurement error of 56 kDa.

For the dot plot, we used a nitrocellulose membrane. we prepared a nitrocellulose membrane (Bio-Rad) and applied TSA56 recombinant protein (*2*) and bovine serum albumin (BSA), the latter serving as a negative control to assess antibody specificity. To ensure non-specific binding was identified, *Rickettsia parkeri* (Rp) protein was used as an unrelated antigen. The assay incorporated positive controls consisting of scrub typhus IgG serum and negative controls of normal human serum. Blocking was conducted using a buffer of 5% non-fat dry milk in PBST (PBS + 0.1% Tween-20) to prevent non-specific binding. The secondary antibody used was HRP-conjugated anti-human IgG (Invitrogen). 1 μg of TSA56 protein, BSA and Rp were spotted onto the membrane, followed by air drying. Serum samples were diluted ranging from 1:100 to 1:100,000 in blocking buffer and incubated on the membrane followed by a 1-hour room temperature incubation. Signal was detected for both the dot plot and the western blot using iBright FL1000 (Thermo Fisher Scientific).

After identification of a reproducible positive RT-PCR result, the *rrs* RNA PCR amplicon from case D was sequenced in both forward and reverse directions. The flanking forward and reverse sequences had high confidence electropherograms to include sequences in both directions for a total high confidence sequence of 96 bp with 46 bp of overlap in both directions. To evaluate the association with the 96-bp sequence from case D with other *Orientia* spp., we performed a phylogenetic analysis of the 96-bp sequence using the Neighbor-Join (NJ) algorithm method using the Biostrings and ape package and figures created using the ggplot2 and ggtree packages in R.

Supplementary Results

We identified evidence of antibody binding against the TSA protein with 6 high titer archived serum samples using a dot blot and with the Western blot in a high titer serum sample. To investigate and validate the findings of seropositivity, we read IFA slides from those that seroconverted with a >1:256 titer (n = 13 including 9 with higher SFG or TG titers excluded from the primary clinical description) and also randomly selected an additional 30 to determine IFA titers. We found that the majority (9 of 13 convalescent samples) of those that seroconverted with the Karp strain also had a Gilliam strain IFA titer of >1:256. The median Gilliam IFA titers among the samples with seroconversion and Karp titers >1:256 was 512 (IQR 32–2,048; range 32–8,192). The Fort Portal participant D with molecularly confirmed *Orientia* spp. infection had an acute serum titer of 1:512 and convalescent serum titer 1:8,192 (16-fold titer change) with Gilliam strain IFA. Among those randomly selected, 2 of 8 acute samples and 1 of 7 convalescent samples with titers >1:256 also had Gilliam strain titers >1:256.

References

1. Blair PW, Kobba K, Kakooza F, Robinson ML, Candia E, Mayito J, et al. Aetiology of hospitalized fever and risk of death at Arua and Mubende tertiary care hospitals in Uganda from August 2019 to August 2020. BMC Infect Dis. 2022;22:869.

2. Mubende Regional Referral Hospital Profile. SUSTAIN Uganda 2018 [cited 2018 Jul 16]. http://sustainuganda.org/sites/sustainuganda.org/files/Mubende%20Regional%20Referral%20Hospital%20Profile.pdf

3. Manabe YC, Betz J, Jackson O, Asoala V, Bazan I, Blair PW, et al. Clinical evaluation of the BioFire Global Fever Panel for the identification of malaria, leptospirosis, chikungunya, and dengue from whole blood: a prospective, multicentre, cross-sectional diagnostic accuracy study. Lancet Infect Dis. 2022;22:1356–64.

4. Sando E, Ariyoshi K, Fujita H. Serological Cross-Reactivity among *Orientia tsutsugamus*hi serotypes but not with *Rickettsia japonica* in Japan. Trop Med Infect Dis. 2018;3:74.

5. Yang S-L, Tsai K-H, Chen H-F, Luo J-Y, Shu P-Y. Evaluation of enzyme-linked immunosorbent assay using recombinant 56-kDa type-specific antigens eerived from multiple *Orientia tsutsugamushi* strains for detection of scrub typhus infection. Am J Trop Med Hyg. 2019;100:532–9.

6. Rasband W. ImageJ version 1.53. Bethesda (MD) USA: National Institutes of Health; 2024.

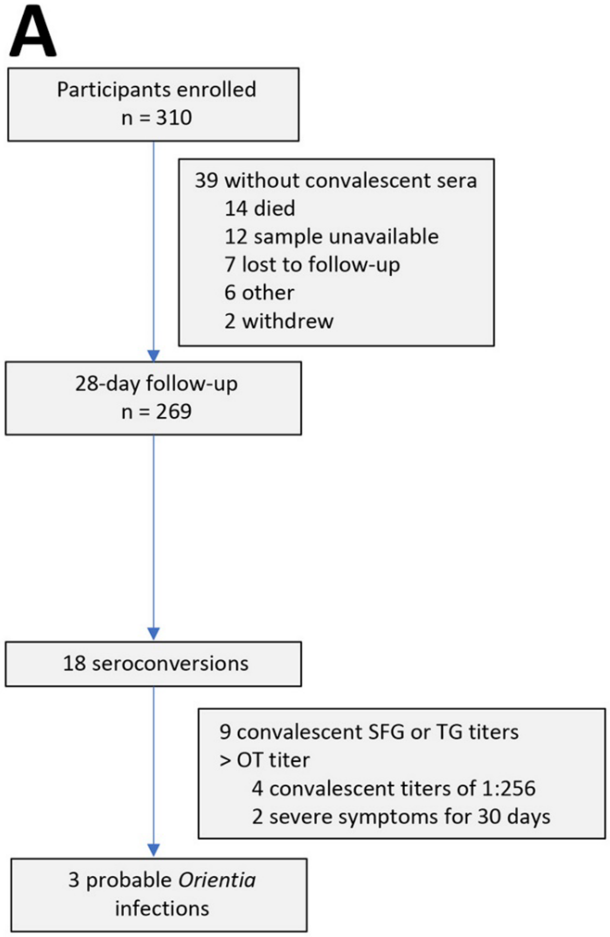

Appendix Figure 1. Flow diagram of patient enrollment and samples used from sepsis (Fort Portal) and acute febrile illness (Arua and Mubende) cohorts, Uganda.

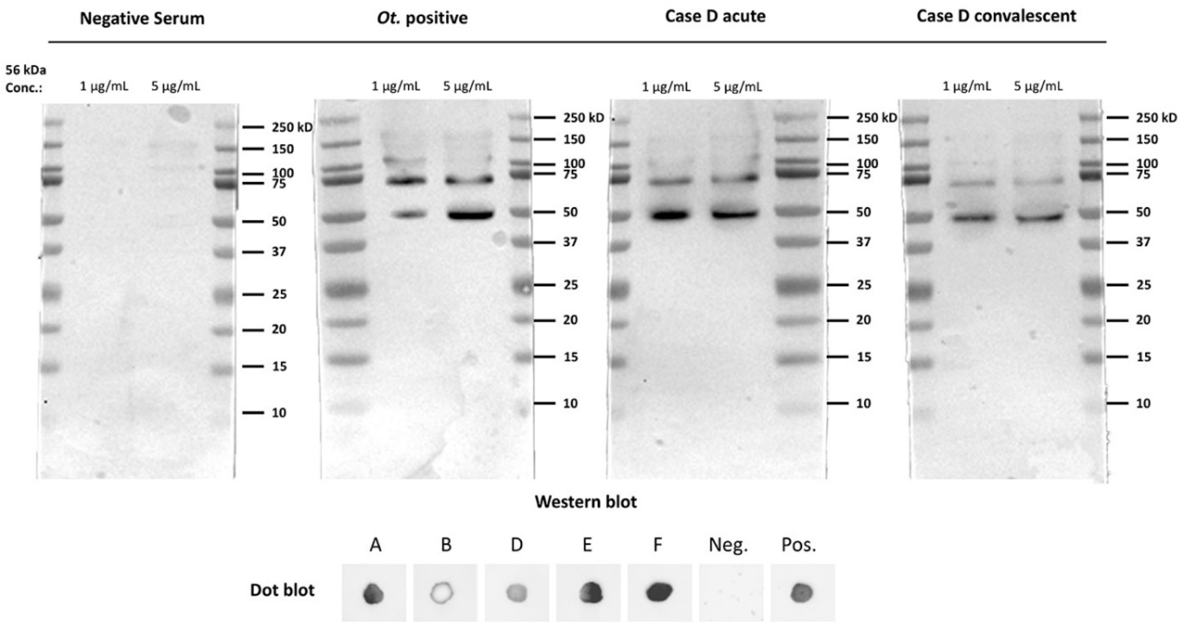

Appendix Figure 2. Western blot identified evidence of a positive band at 56-kDa at two protein concentrations and case D's acute and convalescent serum samples matched those of a positive serologic *Orientia tstutugamushi* control. This was absent in an unexposed healthy serum control. Primary antibodies were diluted at 1:100,000. The dot blot showed evidence of antibody binding with convalescent sera (diluted at 1:1,000) from IFA seroconversion cases A, B, D, and high IFA IgG titer cases E and F. Neg, healthy control serum; Pos, scrub typhus IgG serum positive control.
